# Assessment of gingival characteristics by professional versus smartphone imaging

**DOI:** 10.34172/japid.026.3806

**Published:** 2025-11-27

**Authors:** Mohan Kumar Pasupuleti, Gurramkonda Sirisha, Gabu Pujitha, Gautami Subhadra Penmetsa, Supraja Salwaji, Nissi Gurindapalli

**Affiliations:** ^1^Department of Periodontics, Vishnu Dental College, Vishnupur, Bhimavaram, West Godavari, Andhra Pradesh, India; ^2^Vishnu Dental College, Vishnupur, Bhimavaram, West Godavari, Andhra Pradesh, India; ^3^Department of Oral and Maxillofacial Pathology, Vishnu Dental College, Vishnupur, Bhimavaram, West Godavari, Andhra Pradesh, India; ^4^Department of Orthodontics, Vishnu Dental College, Vishnupur, Bhimavaram, West Godavari, Andhra Pradesh, India

**Keywords:** Colorimetry, Computer-assisted photography, Image processing, Smartphone

## Abstract

**Background.:**

Accurate evaluation of gingival health requires understanding inflammation as a sign of disease activity or healing. Only a few studies have examined the gingiva in depth using non-invasive imaging techniques. Therefore, this study assessed the accuracy of evaluating gingival parameters using professional (Canon EOS 1300D, Canon Inc., Japan) and smartphone (iPhone 15 Pro, Apple Inc., USA) cameras, with pre- and post-treatment photographs.

**Methods.:**

Thirty-four patients with gingivitis were selected, and photographs were captured using professional and smartphone cameras. Gingival parameters were examined using pictures of the maxillary anterior region taken at distances of 24, 28, and 32 cm from the examination site. All the images were evaluated using the free ImageJ software (US National Institutes of Health, Bethesda, Maryland, USA) with an accuracy of 0.01 mm. The paired t-test was used to compare gingival color values and clinical measurements. A P value of<0.05 was considered statistically significant.

**Results.:**

There were no significant differences in gingival parameters between images taken using either professional or smartphone photography. The results showed no significant differences in gingival color assessment at 24-, 28-, and 32-cm distances between the two images.

**Conclusion.:**

Digital images obtained with DSLR and smartphone cameras showed comparable accuracy for gingival parameter measurements at the tested distances.

## Introduction

 The evaluation of gingival health has typically been based on clinical probing, visual inspection, and colorimetric assessment. Currently, clinical probing with a calibrated periodontal probe remains the gold standard for measuring sulcus depth, clinical attachment, and bleeding on probing; however, it is invasive, technique-sensitive, and often uncomfortable for the patient. Visual inspection is non-invasive, clinically user-friendly, and therefore easy for the clinician to identify inflammation, edema, and hypertrophy. Despite being non-invasive, visual inspection remains subjective and less reliable in the early stages of gingivitis, and the diagnosis may very well rely on the examiner’s experience.^1,2^

 To consider quantitative structure, colorimetric assessments similar to all indices, including the modified gingival index (MGI), assess changes in gingival color and texture, which indicate inflammatory status. Although colorimetric indices do provide standardized criteria (e.g., scores based on redness or bleeding), they still rely on clinicians’ interpretation of color and are subject to variability. Other, more objective techniques include transgingival probing (which measures gingival thickness through probe transparency), which is quantitative but invasive, and ultrasound imaging or CBCT; all are objective and provide non-invasive exact numbers but come at a higher cost and are more complex, or, in the example of CBCT, expose patients to radiation.^1,2^

 Dental practitioners must have anatomical knowledge of the periodontium, including its size, composition, and orientation, to understand the pathophysiology and aesthetics of the periodontal and surrounding tissues. The attached gingiva (AG) and keratinized gingiva (KG) exhibit distinct histological and morphological features. Collagen fibers securely link AG to the root cementum and alveolar bone, while KG is a combination of AG and the outer surface of the periodontal pocket or the bottom of the gingival sulcus.^3,4^

 Prior research often employed a caliper and a periodontal probe to measure the gingival dimensions. The state of the periodontium, the expertise level, interference from nearby anatomic structures during measurement, and the set graduations on measuring instruments are among the potential drawbacks of various measurement techniques. The extensive use of digital technology in dentistry today offers a novel opportunity to address the aforementioned constraints.^5,6^

 The assessment of gum health has historically been viewed as a fundamental aspect of comprehensive dental care because of its direct connection to overall oral health and its role in preventing various dental diseases. The gums, known as the gingiva, serve as a protective shield for the teeth and the supporting bone structures, and their well-being is essential for maintaining the functional and esthetic integrity of the mouth.^7-9^

 The advancement of non-invasive methods for evaluating gingival health is a hopeful area of dental research. Historically, assessing gingival health has relied on clinical methods such as visual inspection, periodontal probing, and radiographic imaging, which, while useful, may be invasive, uncomfortable for patients, and prone to error. Furthermore, these traditional procedures need direct contact with the gums, which can cause discomfort (e.g., bleeding or pain while probing), especially in individuals with sensitive or previously inflamed tissues.^9,10^

 Non-invasive diagnostic approaches, on the other hand, have the potential to alleviate some of these limitations by providing a more pleasant, effective, and patient-centered approach to monitoring gingival health. Despite the apparent advantages, research in this field is limited, and the non-invasive approach has not yet demonstrated clinical feasibility. Several factors have contributed to that, including the complexity of gingival disease, limitations in current technology, and the need for a comprehensive evaluation of a new approach.^11-13^

 Thus, digital technologies can improve anatomical information. The goal of the current study was to compare gingival dimensions obtained from smartphone and DSLR (digital single-lens reflex) camera digital photos with those obtained by conventional clinical measurements using probes and by eye inspection for color evaluation.

## Methods

 The protocol of the present cross-sectional observational study was approved by the institutional Ethics Committee of Vishnu Dental College under reference number IECVDC/24/F/PI/IVV/49. The study was conducted between April and June 2024.

 The inclusion criteria for the study were (1) volunteers affiliated with Vishnu Dental College, (2) age of ≥ 20 years, (3) satisfactory oral hygiene, defined by a full-mouth plaque index of < 20% and a full-mouth bleeding index of < 20%, and (4) no missing teeth. The exclusion criteria included (1) a history of orthodontic treatment, (2) mal-aligned dentition, and (3) current periodontal disease or a history of periodontitis.

 In the study, 34 patients diagnosed with gingivitis were selected as participants to investigate their gingival health. Gingivitis is a common, mild form of periodontal disease characterized by inflammation of the gums, usually caused by plaque buildup at the gingival line. If left untreated, it can lead to more serious periodontal disease. The choice of these individuals allows us to concentrate research on gingival problems that are still in the early stages, without causing further damage to tissues found during periodontitis.

###  Patient selection

 The patients were carefully selected based on specified inclusion criteria, which most likely included gingivitis but no other severe forms of gingival disease (such as periodontitis). This guaranteed that the research was limited to early-stage gingivitis, which can be effectively treated or reversed with good dental hygiene. The patients were also required to have acceptable oral hygiene (e.g., low plaque and bleeding indices) to prevent confounding variables in assessing gingival health.

 Gingival clinical parameters and gingival color accuracy were examined using pictures of the maxillary anterior region taken at distances of 24, 28, and 32 cm from the examination site. Patients’ photographs were taken at 24, 28, and 32 cm with a DSLR camera and a smartphone.

###  Photographic image capture and lens selection

 To assess the condition of the patients’ gums, photographs were captured using both professional cameras and smartphone cameras. Photographs were taken using a DSLR camera and an iPhone 15 Pro. Dental photographs were taken in this study with a professional camera using a Canon 18–55 mm kit lens, rather than a macro lens such as the Canon 100 mm macro lens typically recommended for standardized dental photography. This justification is based on availability. Lens selection can affect image magnification, distortion, depth of field, and the reproduction of fine gingival details. We acknowledge that the photographic parameters may have been affected by using a kit lens and will take this potential impact into account when reviewing the results.

###  Lighting conditions

 Intraoral photographs were taken using the camera’s built-in pop-up flash as the primary light source. The flash was positioned directly over the lens to allow for frontal lighting. Due to the lack of directional or diffused lighting, this method can sometimes produce uneven lighting, glare, or shadows, especially from the lips over the maxillary anterior area. There were no external flash units, ring flashes, or diffusers used during this procedure. These lighting conditions were taken into account when interpreting color accuracy and morphometric measurements from the images.

###  Professional camera images

**Figure 1:**
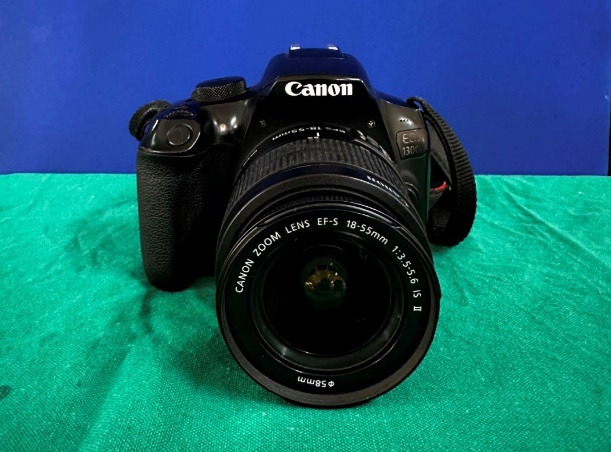
DSLR camera setup used for gingival imaging

 Professional cameras produce high-resolution, complex images and can capture small details of gum inflammation, such as redness, swelling, bleeding, and plaque. They also provide improved control of the lighting that guarantees a homogeneous and transparent image without fluctuating with natural light. Moreover, these cameras provide standardization by ensuring images are captured under ideal conditions, including uniform angles, magnification, and lighting, which is vital for precise, accurate assessments of gingival health (Figure 1).

###  Smartphone camera images

**Figure 2:**
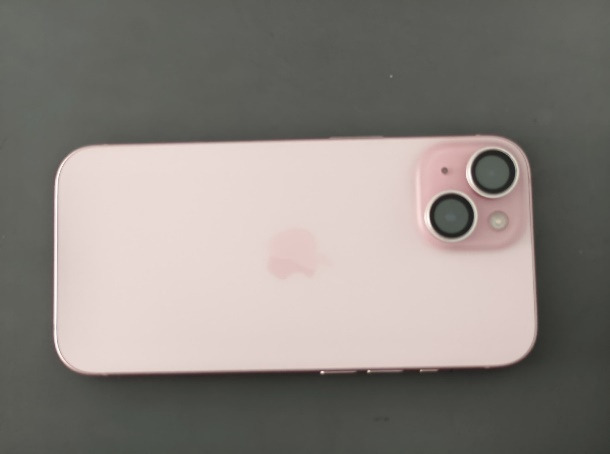
Smartphone setup used for gingival imaging

 Although smartphone cameras usually have lower resolution than professional cameras, their availability and user-friendliness make them useful tools for taking photos in less regulated environments, such as dental offices. Their combination with mobile apps and editing software enables clinicians to conduct real-time evaluations, resulting in speedier documentation and image sharing. Furthermore, smartphone cameras allow for comparison studies to determine whether they can provide adequate image quality for clinical examinations, making them a viable and cost-effective alternative to specialized equipment in medical settings (Figure 2).

 In this study, gingival clinical parameters and gingival color accuracy were examined by capturing photographic images of the maxillary anterior region (the upper front teeth and surrounding gingival tissues) before scaling and root planing, which are commonly performed to remove plaque and tartar from the teeth and below the gumline to treat gingivitis and early-stage periodontitis.

###  Image capture procedure

 Images of the maxillary anterior area were captured at three designated distances from the examination location: 24, 28, and 32 cm. These different distances were selected to examine how the distance between the camera and the subject can affect the quality and precision of captured images. The study examined photos taken from various distances to identify the optimal distance for capturing gingival traits with the most detail and clarity. The decision to record photos at varying distances allows investigation of how distance affects image quality and depth of field, potentially influencing the precision of gingival color identification and the evaluation of clinical parameters (such as gingival redness, swelling, and tissue form).

 Images were taken using both a smartphone and a DSLR camera. The DSLR camera, known for its high resolution and precise control over characteristics such as focus, lighting, and exposure, produces crisp, precise photos. In contrast, the smartphone camera is more convenient and accessible, and its performance was compared with that of the DSLR camera to determine whether it could produce results sufficiently accurate for clinical assessments.

 Traditionally, the assessment of gingival color employed subjective terms, such as “pink” or “coral pink”, for example, which were unstandardized and nonreproducible. These qualitative terms were also subject to the investigator’s interpretation, clinical judgement, and even lighting conditions, which limited their clinical and research value. It was necessary to move to objective colorimetry because of the ability to view colors in different ways, and the CIELab color space (Commission Internationale de l’Éclairage Lab*, or short “CIE”) was a scientifically valid method of assessing gingival color through measurement of color in three axes: L: lightness as our first axis, a: red-green as our second axis, and b: yellow-blue as our third axis. CIELab provided an objective, precise, repeatable, and standardized means of measurement.

###  Post-procedure image examination

 After photo capture, the images were analyzed using ImageJ, software developed by the U.S. National Institutes of Health. ImageJ enables accurate measurement of characteristics such as gingival color, width, and shape alterations. The software delivers measurements with a precision of 0.01 mm, enabling thorough examination of subtle variations in gingival health, including enhancements in gingival color and decreases in inflammation. Using ImageJ, clinicians can objectively measure important clinical factors, such as gingival margins, bleeding on probing, and plaque buildup, which aid in assessing the success of scaling and root planing and in monitoring the extent of gingival inflammation.

 In this research, SPSS was employed to evaluate and contrast clinical data and gingival characteristics captured using professional and smartphone cameras. The objective was to determine whether statistically significant differences existed between the two imaging techniques for evaluating gingival health, particularly gingival color, margins, swelling, and various clinical characteristics.

###  Sample size calculation and statistical analysis

 To compute the required sample size for a paired t-test (dependent means), an a priori power analysis was conducted with the following inputs: a two-tailed test, an effect size of dz = 0.5, an alpha error probability (α) of 0.05, and a desired power of 0.80 (1 - β). With these values, the crucial t-value was found to be 2.0345153, and the non-centrality parameter (δ) was found to be 2.9154759. After determining that there were 33 degrees of freedom (df), a total sample size of 34 individuals was required. This sample size yielded a real power of 0.8077775, which is comparable with the desired power of 0.80 and provides adequate statistical sensitivity to detect the impact. The study sought to statistically assess if there were significant variations in gingival color accuracy and clinical assessments at camera-to-patient distances of 24, 28, and 32 cm.

## Results

 The study used a combination of high-quality photographic imaging and precise image analysis software to assess gingival health and color accuracy before scaling and root planing. By using both DSLR and smartphone cameras and analyzing the images with ImageJ software, the research aimed to determine whether smartphone cameras could be used as a viable alternative to professional equipment in clinical settings for monitoring and evaluating gingival conditions, offering a cost-effective and accessible solution for dental professionals.

 The gingival parameters measured were the width of KG, height of interdental papilla, gingival margin position, and width of attached gingiva at 24-, 28-, and 32-cm distances by using a DSLR camera and a smartphone.

###  Inter-group comparison: camera vs. smartphone at 24, 28, and 32 cm 

 The means and standard deviations (SD) of the width of KG measured digitally by both a DSLR camera and a smartphone were 5.7265 ± .60016 and 5.6118 ± .62122 at 24 cm, 5.5941 ± 0.64099 and 5.4941 ± 0.64099 at 28 cm, and 5.4265 ± .60016 and 5.3941 ± 0.64099 at 32 cm. The t-values and P-values of the width of KG measured digitally by both a DSLR camera and a smartphone were 0.774 and 0.442 at 24 cm, 0.643 and.522 at 28 cm, and 0.215 and 0.831 at 32 cm (Table 1).

 The means and standard deviations (SD) of the height of interdental papilla measured digitally by both a DSLR camera and a smartphone were 2.4824 ± 0.43656 and 2.4088 ± 0.42737 at 24 cm, 2.3824 ± 0.43656 and 2.3088 ± 0.42737 at 28 cm, and 2.2824 ± 0.43656 and 2.2088 ± 0.42737 at 32 cm. The t-values and *P* values of the height of interdental papilla measured digitally by both a DSLR camera and a smartphone were 0.723 and 0.485 at 24 cm, 0.723 and 0.485 at 28 cm, and 0.723 and 0.485 at 32 cm (Table 1).

 The means and standard deviations (SD) of gingival margin position measured digitally by both a DSLR camera and a smartphone were 0.4706 ± 0.78760 and 0.4706 ± 0.78760 at 24 cm, 0.4706 ± 0.78760 and 0.4706 ± 0.78760 at 28 cm, and 0.4706 ± 0.78760 and 0.4706 ± 0.78760 at 32 cm. The t-values and *P* values of the gingival margin position measured digitally by both a DSLR camera and a smartphone were 0.000 and 1.000 at 24 cm, 0.000 and 1.000 at 28 cm, and 0.000 and 1.000 at 32 cm (Table 1).

 The means and standard deviations (SD) of the width of attached gingiva measured digitally by both a DSLR camera and a smartphone were 3.4765 ± 0.47486 and 3.4765 ± 0.47486 at 24 cm, 3.3765 ± 0.47486 and 3.3765 ± 0.47486 at 28 cm, and 3.2765 ± 0.47486 and 3.3059 ± 0.46250 at 32 cm. The t-values and *P* values of the width of attached gingiva measured digitally with both a DSLR camera and a smartphone were 0.000 and 1.000 at 24 cm, 0.000 and 1.000 at 28 cm, and -0.259 and 0.797 at 32 cm (Table 1).

**Figure 3:**
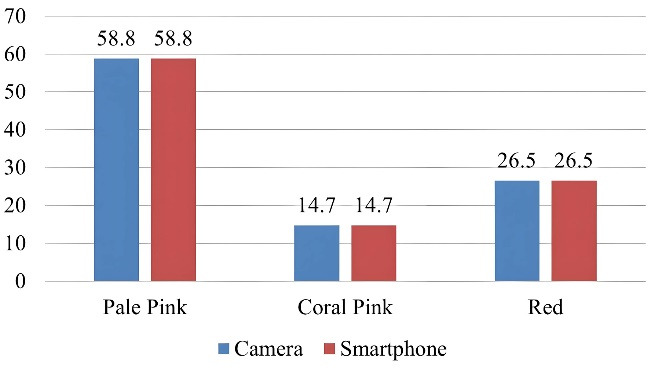
Gingival color comparison between DSLR camera and smartphone at 24, 28, and 32 cm

 The gingival colors assessed digitally with a DSLR camera and a smartphone at 24, 28, and 32 cm were pink (58.8%), coral pink (14.7%), and red (26.5%). There was no difference in color assessment at any of the three distances between the DSLR camera and smartphone sources (Figure 3)(Supplementary file 1).

###  Inter-group comparison: traditional vs. camera vs. smartphone 

 No statistically significant differences were observed in gingival characteristics measured by traditional clinical exam, professional camera imaging, and smartphone imaging (Supplementary file 2).

*Width of KG:* The mean widths measured by traditional clinical examination (5.70 ± 0.56 mm), professional camera imaging (5.73 ± 0.60 mm), and smartphone imaging (5.61 ± 0.62 mm) were very similar (*P* = 0.442), indicating that both imaging modalities can consistently reproduce the traditional clinical measurement of KG width (Table 2).

*Height of interdental papilla (IDP):* The height measurements followed a similar pattern with means of approximately 2.4–2.6 mm for all methods and no significant differences (P = 0.485). This indicates that imaging methods can determine IDP height (Table 2).

*Gingival margin (GM) position:* The mean GM position value was the same for camera imaging (0.47 ± 0.79) and smartphone imaging, and comparable to the standard method, with a P-value of 1.000, which indicated perfect agreement (Table 2).

**Figure 4:**
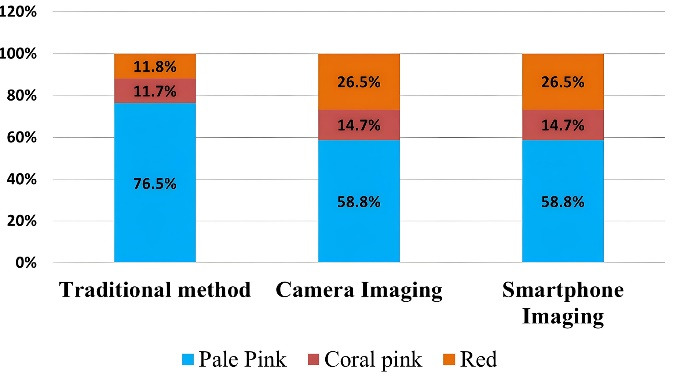
Gingival color comparison between the traditional method, DSLR camera, and smartphone imaging

**Figure 5:**
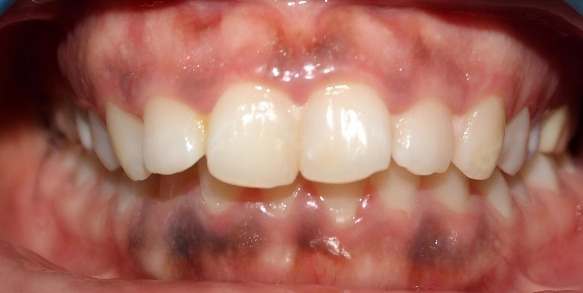
Gingival color assessment using a DSLR camera at different examination distances

**Figure 6:**
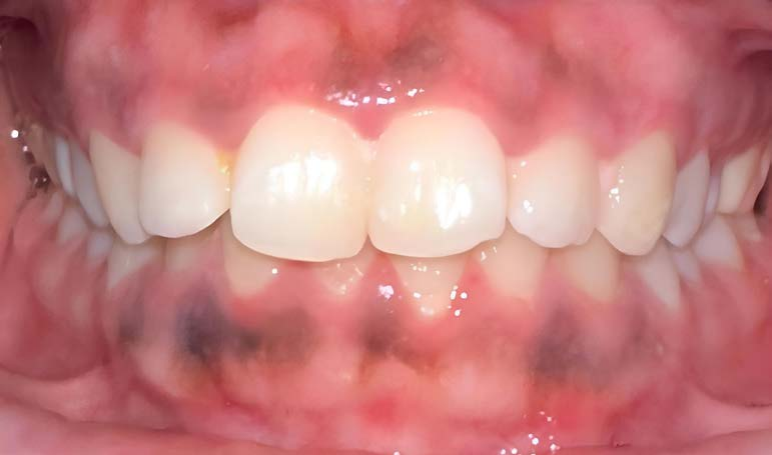
Gingival color assessment using a smartphone at different examination distances

*Gingival color:* Among the categories (pale pink, coral pink, red), the proportions of gingival color were not significantly different across the three methods used (*P* = 0.185). Although the proportion of pale pink gingiva was larger in the traditional method group (76.5%) than in the two imaging methods (58.8%), the difference was not statistically significant. Participants demonstrated a consistent ability to assess gingival color using both camera and smartphone imaging (Table 2 and Figure 4). Gingival color assessment in pictures captured with a camera and a smartphone was performed using the standardized CIELab color measurement method, yielding precise, standardized results (Figures 5 and 6).

**Table 1:** Comparison of the mean ± SD of the width of keratinized gingiva, height of interdental papilla, gingival margin position, and width of attached gingiva at 24, 28, and 32 cm using DSLR and smartphone cameras

**Parameter**	**Group**	**N**	**Mean (mm)**	**SD**	**t-value**	***P*** ** value**
Width of keratinized gingiva (KG) at 24 cm	DSLR camera	34	5.73	0.60	0.774	0.442
	Smartphone camera	34	5.61	0.62		
KG at 28 cm	DSLR camera	34	5.59	0.64	0.643	0.522
	Smartphone camera	34	5.49	0.64		
KG at 32 cm	DSLR camera	34	5.43	0.60	0.215	0.831
	Smartphone camera	34	5.39	0.64		
Height of interdental papilla (IDP) at 24 cm	DSLR camera	34	2.48	0.44	0.723	0.485
	Smartphone camera	34	2.41	0.43		
IDP at 28 cm	DSLR camera	34	2.38	0.44	0.723	0.485
	Smartphone camera	34	2.31	0.43		
IDP at 32 cm	DSLR camera	34	2.28	0.44	0.723	0.485
	Smartphone camera	34	2.21	0.43		
Gingival margin (GM) position at 24 cm	DSLR camera	34	0.47	0.79	0.000	1.000
	Smartphone camera	34	0.47	0.79		
GM at 28 cm	DSLR camera	34	0.47	0.79	0.000	1.000
	Smartphone camera	34	0.47	0.79		
GM at 32 cm	DSLR camera	34	0.47	0.79	0.000	1.000
	Smartphone camera	34	0.47	0.79		
Width of attached gingiva (AG) at 24 cm	DSLR camera	34	3.48	0.47	0.000	1.000
	Smartphone camera	34	3.48	0.47		
AG at 28 cm	DSLR camera	34	3.38	0.47	0.000	1.000
	Smartphone camera	34	3.38	0.47		
AG at 32 cm	DSLR camera	34	3.28	0.47	−0.259	0.797
	Smartphone camera	34	3.31	0.46		

KG: Keratinized gingiva; IDP: Interdental Papilla; GM: Gingival margin; AG: Attached gingiva. The t-value quantifies how large the difference between group means is relative to sample variability, while the p-value shows the probability of observing such a difference (or a more extreme one) if the null hypothesis were true—typically, *P* < 0.05 indicates a statistically significant result.

**Table 2:** Comparison of gingival parameter measurements using the traditional periodontal probe method (group 1), DSLR camera method (group 2), and smartphone method (group 3)

**Parameter**	**Traditional method (n=34) Group 1**	**Camera imaging** **(n=34) Group 2**	**Smartphone imaging** **(n=34) Group 3**	***P*** ** value**
Width of keratinized gingiva (KG) (mm)	5.70 ± 0.56	5.73 ± 0.60 (at 24 cm)	5.61 ± 0.62 (at 24 cm)	0.442
Height of interdental papilla (IDP) (mm)	Mode = 3; Mean ≈ 2.63	2.48 ± 0.44 (at 24 cm)	2.41 ± 0.43 (at 24 cm)	0.485
Gingival margin (GM) position (mm)	Mostly 0; few cases with 1 or 2	0.47 ± 0.79	0.47 ± 0.79	1.000
Gingival color	26 Pale pink (76.5%) 4 Coral (11.8%) 4 Red (11.8%)	20 Pale pink (58.8%) 5 Coral (14.7%) 9 Red (26.5%)	20 Pale pink (58.8%) 5 Coral (14.7%) 9 Red (26.5%)	0.185

 These results show that smartphone imaging is a tool comparable to professional camera imaging and traditional clinical assessment for determining key gingival attributes such as width, height, margin position, and color. Therefore, smartphone imaging could be used as a reliable, accessible alternative method for clinical and research applications when assessing gingival characteristics.

## Discussion

 This study aimed to compare the accuracy and precision of smartphone imaging with DSLR photography in assessing gingival characteristics. Although initial data on smartphone vs. DSLR imaging showed potential for clinical use, evaluations aimed at a deeper understanding of the implications are warranted to integrate the data into broader clinical literature.

 Technically, the assessment of focusing and distance estimation in the work of Healy and Stephan advances our understanding of smartphone vs. DSLR photography, with clearly identified limitations related to device-specific variance and experience.^10^ Controllers of device and user experience were clearly needed, which necessitate operational processes, calibration devices, and potentially software enhancements (i.e., AI computer-aided corrections) to ensure consistency for smartphone imaging.

 Recent research demonstrated that smartphones are increasingly feasible for clinical photography, and Yung et al^14^ noted that new smartphone cameras can achieve color accuracy similar to that of a DSLR system in a controlled clinical setting. They recommended smartphones for intraoral photography, provided that user protocols for lighting and positioning are followed. This is consistent with our results, which showed that smartphone photography can reasonably represent gingival color and contour.

 Additionally, Lazar et al^15^ explored the use of smartphones for aesthetic evaluation in dentistry and concluded that smartphones could be appropriately used for preliminary aesthetic evaluations, particularly in low-resource environments or for patient self-assessment. Our results endorse this use, especially for screening or follow-up purposes, although professional systems provided more detailed images with more consistent image quality across different lighting conditions.

 Gingival health and color consistency were assessed following scaling and root planing using high-resolution digital imaging and precise image processing techniques. The study seeks to determine whether smartphone cameras can serve as a cost-effective, easy alternative to professional devices for diagnosing and monitoring gingival diseases in clinical settings.^13-15^

 The use of DSLR and smartphone cameras enables a comparison of their performance in terms of color correctness, resolution, and gingival parameter evaluation. The use of ImageJ software for image analysis ensures impartiality in image analysis, providing a consistent way to measure and compare gingival health. This method is clinically relevant because it allows for non-invasive monitoring of gingival health using pre- and post-treatment photographs, allowing clinicians to track improvements in gingival color and other key parameters following scaling and root planing, assisting in the evaluation of treatment effectiveness.^16,17^

 Moussa et al^18^ conducted a study to compare linear measures of clay models taken with DSLR and smartphone cameras to digital models. The measures of anterior teeth taken with DSLR and smartphone cameras (at all distances tested) and scanned showed no difference. For documentation reasons, distortion is minimal, and both camera systems can be used. Dentists can use DSLR and smartphone cameras (at a minimum distance of 24 cm) to capture smiles and provide comparable, consistent linear measurements.

 Lim et al^19^ conducted a study to revisit the gingival dimensions in a healthy Korean population using digital scanning. According to the study’s findings, gingival dimensions obtained with an intraoral scanner followed a distribution pattern similar to those found in earlier investigations. However, the proportions appear to be affected by race and/or ethnicity, particularly in the mandibular canine and second molar. Some gingival measurements may be used to differentiate between males and females.

 A thorough evaluation of the relationship between periodontal phenotype and gingival dimensions found that the mean KG height ranged between 2.75 and 5.44 mm in the thin phenotype and 5.09 mm to 6.65 mm in the thick phenotype. In the current investigation, the mean keratinized gingival widths measured by DSLR and smartphone cameras at a distance of 24 cm were 5.33 mm and 5.7 mm, respectively.^20^

 Bhatia et al^21^ conducted a study to measure the entire-mouth mid-buccal breadth of attached gingiva in individuals across four distinct age groups. This study also examined differences between optical and histochemical approaches for identifying the mucogingival junction and for calculating the breadth of the attached gingiva. The study’s findings revealed that the width of attached gingiva varies in different regions of the mouth and increases with age, with no significant differences in the evaluation technique. The present study results are also consistent with this study, which employed both DSLR and smartphone cameras to examine gingival features with no significant difference in the mode of photography.

 Kuppusamy et al^22^ conducted a study to assess the accuracy of smartphone photos for dental health screening in children compared with clinical examination. The pilot study found that a mobile teledentistry technique using smartphone photos can diagnose caries, plaque, and gingival status in children, with diagnostic accuracy comparable to that of a visual clinical examination. Compared with a visual clinical examination, smartphone cameras can be a more reliable and convenient option for screening for enamel and dentin carious lesions, dental plaque, and gingival health. The current study’s findings are also consistent with Kuppusamy et al’s^22^ study, which employed both DSLR and smartphone cameras to examine gingival features with no significant difference in the photographic approach.

###  Summary and outcome of the study

 The clinical measurements, such as the width of KG, attached gingiva, interdental papilla height, gingival margin position, and gingival color, were accurate at 24 cm with both DSLR and smartphone cameras, compared with the traditional clinical measurements using a probe and color assessment by visual examination.

 Using DSLR cameras and smartphones to assess clinical parameters in patients with gingivitis offers numerous benefits for documentation, diagnosis, patient involvement, and remote treatment. Nonetheless, it is important to thoroughly assess image quality, consistency, and data protection.

 Despite considerable interest in developing non-invasive approaches to assess gingival health, existing research remains somewhat limited. Advancements in optical imaging, salivary biomarkers, and diverse diagnostic technologies may revolutionize the assessment of gingival health, reducing patient discomfort and enhancing the accuracy and accessibility of early disease identification.

###  Potential clinical impact and future research directions

 This study has important clinical implications by assessing the reliability of smartphone-based imaging for objectively evaluating gingival characteristics compared with professional equipment. If smartphone-based imaging is validated. Mobile phone imaging could make oral health monitoring accessible to everyone by enabling remote, inexpensive, and cost-effective assessments of periodontal disease, particularly in disadvantaged or low-resource populations.

 While using an 18–55-mm kit lens in place of a dedicated macro lens could affect the measurement accuracy and comparability of the present study relative to previous studies, dedicated macro lenses are designed to provide greater magnification with minimal distortion. Specifically, where kit lenses may introduce slight distortion and a decrease in depth of field, this may impact reproducibility. In light of these limitations, one could argue that this may exacerbate the perceived differences in the quality of professional images compared to smartphone images; therefore, the equivalence of image findings should be taken with caution.

 Future studies should consider developing an imaging protocol standardized across multiple smartphone models, using color calibration devices, and developing computerized diagnostic support systems powered by artificial intelligence to increase the accuracy of the resulting images. Additionally, longitudinal studies could assess the feasibility of using mobile imaging to track disease progression or treatment outcomes in periodontal patients.

 Future research might mitigate this impact by using standard external light sources, e.g., ring flashes and dual-point flashes, which provide more uniform illumination and reduce opportunities for shadows or glare on surfaces.

## Limitations

 This study was limited by potential variability in lighting conditions, camera resolution, and image capture angles, which may have influenced the accuracy of gingival parameter measurements. Differences in operator handling and ambient illumination could also affect image consistency between smartphone and professional camera recordings. Additionally, the study’s sample size and controlled clinical setting might not fully represent real-world conditions.

## Conclusion

 The present study demonstrates that non-invasive digital imaging can reliably quantify gingival clinical characteristics. Smartphone imaging produced measurements comparable to those obtained with a professional DSLR camera across key parameters, including the width of KG, the height of the interdental papilla, the position of the gingival margin, and gingival color. These findings indicate that smartphone-based imaging is a feasible and accessible alternative for assessing gingival health, particularly in settings where professional photographic equipment may not be available. Integration of digital imaging into clinical practice may enhance documentation, facilitate patient education, and support consistent follow-up evaluations.

## Competing Interests

 The authors declare that they have no competing interests.

## Data Availability

 The data are presented as supplementary files.

## Ethical Approval

 All the procedures were in accordance with the ethical standards of the National Committee on Human Experimentation and with the Helsinki Declaration of 1975, as revised in 2013. The study was explained to patients, and a detailed informed consent was obtained from all the participants. The study was approved by the Institutional Ethics Committee of Vishnu Dental College, Bhimavaram, Andhra Pradesh, India, with the reference number IECVDC/24/F/PI/IVV/49.

## Supplementary Files


Supplementary file 1. Data obtained in the research.

Supplementary file 2. Excel sheet of Figures 3 and 4.
